# Telemetry and Sensing Using a Dual-Element Implantable MIMO Antenna System

**DOI:** 10.3390/s26051694

**Published:** 2026-03-07

**Authors:** Amor Smida

**Affiliations:** Department of Medical Equipment Technology, College of Applied Medical Science, Majmaah University, Al-Majmaah 11952, Saudi Arabia; a.smida@mu.edu.sa

**Keywords:** wireless capsule, stomach, implantable antenna, link margin, gain, tumor

## Abstract

Diseases of the gastrointestinal tract (GI) represent a major global health burden, leading to more than eight million deaths each year, largely driven by malignant conditions such as cancers and tumors. Early detection of such conditions can significantly improve survival rates. In this work, we present a compact two-port MIMO topology for high-speed telemetry and sensing. This system integrates two identical antennas, each operating at 915 MHz, positioned only 0.55 mm apart. It has just 11.9 mm^3^ (6.9 mm × 6.9 mm × 0.25 mm) volume, achieved through the use of meandered resonator and a high-dielectric laminate for miniaturization. Despite its small size, the design delivers a measured peak gain of −25.1 dBi at resonance. Low mutual coupling in the antenna-system is made possible by maintaining an optimized spacing and introducing a slot in the ground plane, resulting in isolation levels above 27.9 dB. The MIMO configuration was evaluated using standard performance metrics, and at an SNR of 20 dB, the system reached a better performance than single-element antenna. Beyond communication, this design also functions as a sensor, with its resonant frequency shifting in response to changes in the surrounding tissue’s permittivity: enabling real-time monitoring of internal physiological changes. Throughout the sensing process, the design maintains good gain and impedance matching, making it a strong candidate for biomedical implants.

## 1. Introduction

Today, medical technology is considered an important base of modern healthcare and provides valuable assistance for diagnosing illnesses and tracking biological processes [1,2,3]. Miniature devices have found their use in different applications, including the design of pacemakers for heart support [4] and the development of capsule endoscopy systems for imaging the GI [5]. Other applications are tongue-operated assist systems [6] and glucose measurement systems [7]. Regardless of their use, these miniature devices have circuitry, power cells, and antennas.

A clear example is the wireless capsule endoscope, which transmits live images and videos as it travels through the GI. Housed inside its capsule casing are a transparent dome, CMOS image lens, several sensors, a battery, and electronics. Because these capsules often remain inside the body for hours, efficient power management is essential. Battery life is dictated largely by the power draw of the internal electronics, and sensing modules are among the more power-hungry components. One way to reduce this burden is to integrate sensing capability directly into the antenna, allowing it to handle both data transmission and physiological monitoring without additional hardware.

Several recent studies have explored this concept of “antenna-sensors” that combine wireless communication with sensing. In [8], a compact design for detecting electrical changes in breast tissue was reported, with a volume of just 13.2 mm^3^ and operation at 2.45 GHz. Functional validation of the sensor was done by tracking changes in resonant frequency due to changes in the dielectric constant of the tissue, which is indicative of tumor growth. For every 10 units of change in permittivity, there was a 9–18 MHz change in resonant frequency. Similarly, in [9], the same idea was used for implants in GIs. In their design, only 2.97 mm^3^ was utilized, and it provided high gains and radiation efficiency. Their method consists of three stacked layers that contain both patches and the ground plane. It was tested using minced pork meat, and it was able to track changes in tissue properties. Both RF sensors rely on the same operating mechanism in which the surrounding biological tissue behaves as a capacitive medium. A reduction in tissue permittivity lowers the effective capacitance and increases the operating frequency. Although very efficient in sensing functions, a disadvantage of these designs is the use of single port communication only, which restricts the channel capacity and leads to multipath and lacks diversity. These issues cause serious problems for present-day implant devices involving high-resolution cameras requiring data rates beyond 78 Mbps [10,11].

MIMO antennas have been proposed for this purpose with the benefit of increased data rates without using extra bandwidth [12,13,14,15,16,17]. Nonetheless, the use of MIMO arrays entails the use of several interconnected antennas whose design makes the overall implant larger [18,19,20]. For example, in [12], compact implantable antenna designs with increased isolation for high-speed telemetry were proposed with the help of a neutralization line. Another design for an implanted MIMO antenna with higher bandwidth was presented in [13], where vias were used for isolating four active MIMO antennas. Another approach to improve radiation isotropy avoids the use of a MIMO array and instead focuses on keeping the implant fully compact to enable easier implantation. This technique can be very useful for monitoring applications and in systems implanted in a biologically sensitive area like the scalp, where the tissue conductivity is low. In improving the isolation in the implanted system and using the electromagnetic principles of biologically sensitive regions like the above-mentioned scalp areas, higher data transfer rates can be supported. This is a very attractive technique for an implanted system, especially for patient monitoring and health care applications involving a required channel link. In [17], an antenna operating at 2.45 GHz used electromagnetic bandgap (EBG) structures to improve isolation. Other designs include a wideband MIMO antenna [18], a compact two-element 433 MHz antenna using capacitive slot loading [19], and a miniaturized MIMO structure with dual meandered resonators [20]. The authors of [21] have designed a dual-band implantable antenna based on meandered geometry. It works at 915 MHz and 2.4 GHz with very small geometry of 9 mm^3^.

Further innovations include a multi-port design with extremely low interference (−35.85 dB at 915 MHz and −31.6 dB at 2.45 GHz) achieved through a thin substrate and discrete inductor [22], and a three-dimensional MIMO antenna designed for moving implants, operating at both 915 MHz and 2.45 GHz with orthogonal element placement to enhance isolation [23]. In [24], a meandered resonator-based MIMO system was reported, maintaining strong gain and efficiency. Although these MIMO designs achieve excellent communication performance, none integrate sensing capabilities. Existing studies generally fall into two distinct groups: single-port antenna–sensor designs that offer restricted data throughput, and MIMO antenna systems that focus on communication performance but do not incorporate sensing capabilities.

To overcome these limitations, this study introduces a novel antenna with sensing and telemetry ability. It integrates high-speed communication and tissue sensing by employing two compact implantable antenna elements. The design integrates two identical antenna radiators (placed apart by 0.55 mm) and occupies a total volume of 11.9 mm^3^ (6.9 mm × 6.9 mm × 0.25 mm). Miniaturization is achieved through a meandered resonator and a high-permittivity substrate, while maintaining a measured peak gain of −25.1 dBi at resonance. High inter-element isolation, exceeding 27.9 dB, is realized by optimized spacing and the introduction of a ground-plane slot. Beyond communication, the proposed structure inherently supports tissue sensing by exploiting shifts in resonant frequency caused by variations in surrounding tissue permittivity. The antenna preserves good impedance matching and stable gain across the sensing range. These features enable simultaneous high-capacity wireless communication and real-time physiological monitoring within a single compact implant, making it suitable for next-generation biomedical devices.

## 2. Design Methodology for the Proposed Antenna System

**Figure 1:**
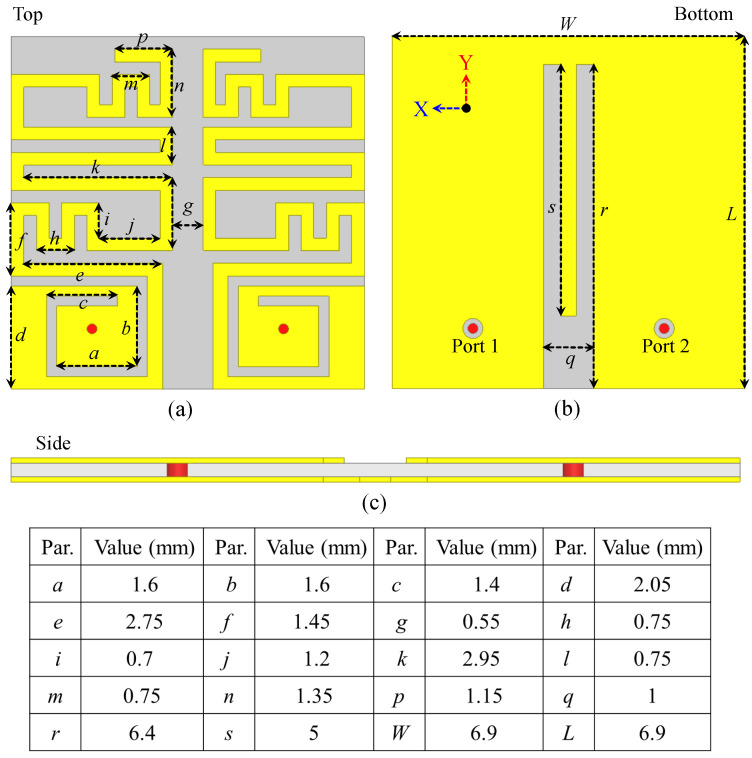
Configuration, layout, and dimensions of the final and optimized design: (**a**) Top view, (**b**) Bottom view, and (**c**) side view.

The optimized MIMO design has two small antenna units, with each antenna designed in a meandered manner to accumulate large currents in a confined region. The final and optimized model is presented in Figure 1. The dimensions of the design are also shown in the same image. These antennas are designed on a Rogers 3010, which has several excellent attributes. A few of them are less losses and high-permittivity. High-permittivity helps in creating a small antenna and less losses helps in better efficiency. The radiator structures lie on the top side, and the reference plane is on the bottom side. These two antennas are facing each other with a distance of 0.55 mm between their edges. The whole antenna-sensor is very small, with a size of 6.9 × 6.9 × 0.25 = 11.9 mm^3^. This small size is possible because of the meandered shape and the high-permittivity substrate. Each antenna is connected using a coaxial probe. The spiral and meandered shapes, along with the high dielectric constant, help keep the design compact.

### 2.1. Implant Placement Within the Simulated Environment

The surrounding of the antenna can also influence the impedance matching, resonant frequency and far-field attributes. Every antenna is mounted in a different location and position based on the device location. Thus, it is always preferable to run the final design in the exact surrounding where it is going to be used. It is also useful to add dummy or actual device components around the antenna and check the influence of these components.

In this paper, proposed design is for a wireless capsule endoscope which is meant for the stomach of a subject. The purpose of the wireless capsule endoscope is to communicate data and recordings from subject to external nodes. In the beginning, the stomach model is imported into the High-Frequency Structure Simulator tool. Afterward, dielectric constant, loss tangent and conductivity are provided to the human body model (100 mm × 475 mm × 740 mm) using information from [25]. The antenna was placed at an implantation depth of 50 mm. The table below (Table 1) gives the precise tissue properties at various frequencies.

**Figure 2:**
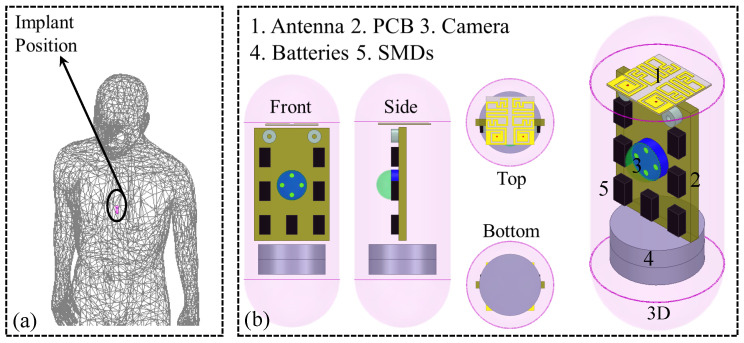
(**a**) Placement of antenna, positioned in the human model (100 mm × 475 mm × 740 mm). (**b**) Structural layout of device, illustrating the integration.

Next, it was positioned inside the stomach to optimize it, as illustrated in Figure 2a. To reach the goal, several optimization steps are done. Parameters such as line width/length, resonators’ separation, and grounded-slot’s dimensions are changed step-by-step using the optimization tools in HFSS. The simulation uses terminal mode and 401 data points. The interpolating sweep method is chosen, and a fine mesh is used for more accurate simulations and meshing.

### 2.2. Structure and Components of the Dummy Implantable Device

The design and functionality of a radiator depend on its shape, the printed circuit board (PCB), nearby device components, and the outer casing. Where these parts are placed can either help or harm how well the antenna works. For instance, having a larger copper layer under the antenna can boost its signal strength. Because of this, it is important to carefully adjust the antenna’s position within its surrounding parts.

These implantable devices include several components. The major ones include but are not limited to PCB, transparent capsule dome, CMOS lens, and batteries. So, the system is designed and tested with all these elements in place. The dummy device designed for this study is illustrated in Figure 2b. All parts are housed inside a dummy device which is designed using polylactic acid (PLA). This material was chosen because it is safe for use in the body, an important factor for medical devices. The complete implant measures 24 mm (length) and 5.75 mm (radius). The casing of the capsule is 0.25 mm thick.

### 2.3. Step-by-Step Development of a Single Radiator

**Figure 3:**
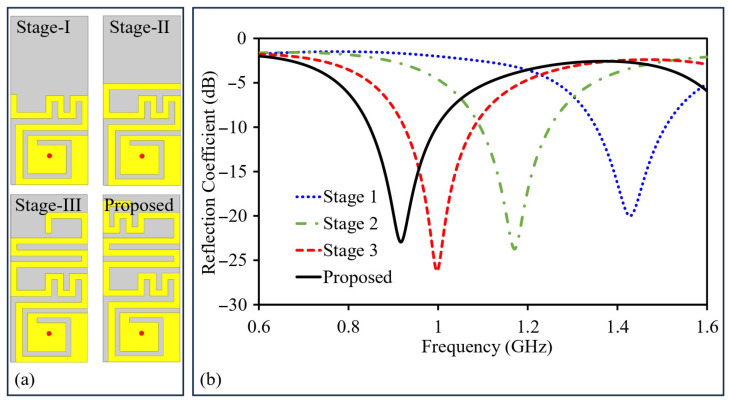
(**a**) Single radiator: design optimization process flow. (**b**) Single radiator: optimization process flow results.

The optimized model of the proposed design contains two identical radiators. Prior to moving on to building and testing the antenna, it is first simulated and adjusted to improve its performance. The process starts with designing and finalizing a single antenna unit. Several steps are taken to make sure it works well (Figure 3). Once the single antenna is optimized, two units are combined to create a MIMO antenna system. During this process, key factors like the radiators’ separation and grounded-slot’s dimensions are carefully adjusted. The main objectives were to keep S_11_ and S_22_ matched over target frequency range, ensure good bandwidth, and maintain low coupling between the antennas. The detailed optimization step-by-step progress can be seen in this section.

First, a section of laminate is created. The bottom ground plane was covered with complete copper. Next, a radiator is placed over the laminate. To power the spiral and meandered radiator, a coaxial feed is adopted. This specific shape is considered because it allows for a long radiator to fit into a small space, which helps to achieve a lower resonance frequency in a compact design. This results in a smaller, miniaturized antenna. The starting design values are derived from

(1) $$L_{res}=\frac{c}{4\times f\sqrt{\epsilon_{r}}}$$

In the first iteration, there exists a spike at a frequency of 1.43 GHz, along with a matched reflection coefficient. In the second iteration, a meandered design is added to the termination of the spiral and meandered structure, which extends the effective electrical path. As a result, the resonant frequency was decreased. The closely spaced sections of the meandered resonator introduce additional capacitive coupling, contributing towards decreasing the overall size of the antenna by decreasing its resonant frequency. The influence caused by this capacitance change on the resonance can be analyzed by examining

(2) $$v_{p}=\frac{1}{\sqrt{L_{res}C_{res}}}=\frac{v_{c}}{\sqrt{\epsilon_{eff}}}=f\lambda_{g}$$

here, $v_{p}$ is propagation velocity, $L_{\mathrm{res}}$ is the effective inductive component and $C_{\mathrm{res}}$ is the effective capacitive component of the radiating structure. It is evident that to achieve compactness, there should be larger effective capacitive components or effective inductive components in the radiating portion of the antenna. Additional capacitance introduced due to the meandered resonator increases the overall reactance, thus achieving antenna size reduction. The near arms in the meandered resonator introduce an extra reactive component, which lower-shifts the operating frequency, and hence overall size reductions are achieved due to larger overall reactance.

In the second stage, the resonance shifts to 1.17 GHz, and the reflection coefficient is approximately −23.68 dB. On progressing to the third stage, an additional meander resonator is incorporated in the design. This further raises the length and shift resonance due to inverse proportionality of frequency and length. Additionally, the meandered shape adds more capacitance, which further reduces the resonant frequency as explained by the slow-wave effect.

In the final stage, the length of the antenna is stretched to achieve resonance at 915 MHz. This design shows BW = 148 MHz and reflection coefficient of –22.92 dB.

Afterwards, a full MIMO antenna is developed. For coupling reduction, grounded-slot was added. In fact, such an approach is most useful when considering a small implantable device where components are packaged tightly alongside one another. In the absence of isolation, the current finds easy passage from one antenna to another using the ground plane. Such a phenomenon is referred to as mutual coupling, and its effect is that it compromises the performance of the device, causing interference and distortion of the entire radiated pattern. Slotted configuration in ground prevents flow of such currents, thus reducing the interference between the antennas. In this approach, there is no need for additional decoupling components, making it most effective within a small biomedical implant where biocompatibility is of significance.

## 3. Results and Discussions

The design was assessed by a two-step process consisting of numerical analysis and experimental verification. The analysis was undertaken by first designing a single antenna-sensor component. Upon satisfying the performance requirements, a second identical component was added to create the proposed design. The two components were placed with a spacing of 0.55 mm. To mitigate mutual coupling, a slot was etched in the ground.

After finishing the design and optimization process in a simulated phantom setup, a physical prototype antenna was developed. The antenna was developed using a Rogers 3010 substrate and a copper metallization layer. After creating both layers of copper, a laser cutting process was used to fabricate the antenna design details. Coaxial cables are soldered to ports of the antenna for easy testing and measurement.

Apart from dedicated assessment, the antenna-sensor was combined in a capsule setup. At the beginning, computer simulations were conducted with the antenna included in the virtual model of the capsule. Subsequently, the combined antenna-sensor was incorporated in a 3D-printed capsule, including a miniaturized PCB and batteries to realistically approximate an implant scenario. In an effort to simulate an in-body scenario, the packaged capsule was buried in meat, a material that has a dielectric constant similar to that of human tissue. These properties were checked using a vector network analyzer to make sure they matched the simulation conditions. After confirming this, measurements were carried out. Before taking any measurements, all the testing equipment was carefully calibrated to ensure accurate results.

**Figure 4:**
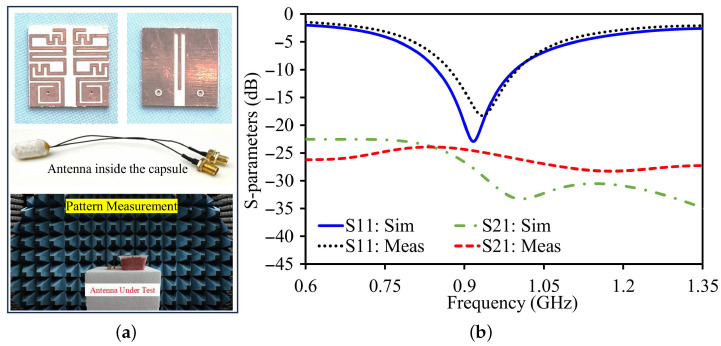
(**a**) Fabricated antenna. (**b**) Antenna simulation and measured data validation.

The S-parameters were characterized using VNA. S_11_ and S_22_ and the coupling between the two antennas (S_21_) were recorded, as shown in Figure 4. In the simulations, the antenna’s resonance for S_11_ and S_22_ occurs at 0.915 GHz, with a bandwidth of 148 MHz, ranging from 0.848 GHz to 0.966 GHz. At this resonance, the simulated reflection coefficients for both S_11_ and S_22_ are about −22.92 dB. The isolation between the two antennas is greater than 27.9 dB within this frequency range.

During the actual measurements, the resonance for S_11_ was found slightly higher, at 930 MHz. The measured bandwidth was 129 MHz for S_11_. The reflection coefficients at resonance were measured as −18.3 dB for S_11_.

**Figure 5:**
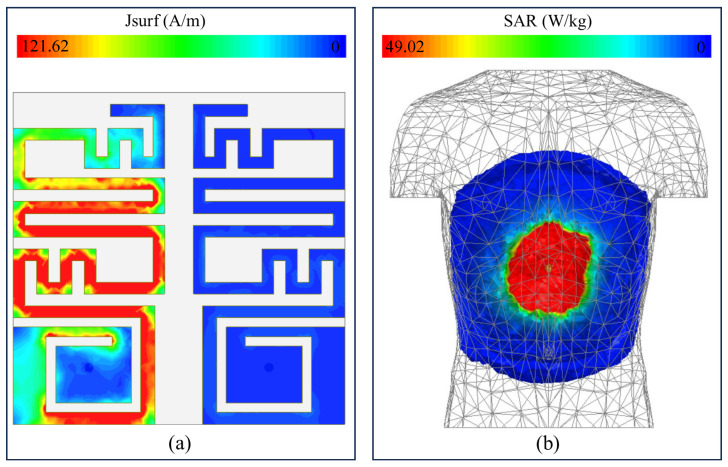
(**a**) Distribution of surface current at 915 MHz and (**b**) corresponding specific absorption rate (10 g) evaluated at 915 MHz.

Figure 5a shows the field distribution of the MIMO sensor. During this analysis, one element was excited with an input power of 1 W, while the second element was kept inactive. The results indicate that the induced current is strongly concentrated on the excited element, whereas only a negligible current appears on the inactive element. This behavior confirms strong electromagnetic isolation between the two units. The achieved isolation is primarily due to the optimized spacing between the resonators, the use of an ultra-thin laminate, and the presence of a ground-plane slot that suppresses surface current coupling.

In addition to achieving good impedance matching and stable radiation behavior, compliance with electromagnetic safety limits within the human body is a critical requirement for implantable systems. In such devices, antennas are used to transmit information from inside the body to an external receiver by radiating electromagnetic energy [26,27]. Part of this energy is absorbed by surrounding biological tissues, and excessive absorption can lead to tissue heating and potential damage.

To quantify this effect, SAR is used as the standard metric for human exposure to electromagnetic fields [28]. International safety guidelines define maximum allowable SAR levels for safe operation, with a commonly adopted limit of 2 W/kg [29,30]. In this work, SAR is evaluated with both antenna ports simultaneously excited. At an operating frequency of 915 MHz and an input power of 1 W, the peak 10 g SAR reaches 49.02 W/kg (Figure 5b).

Due to the linear dependence of SAR on power, it is scaled by this value for realistic working conditions. In practical applications, transmit power is restricted to −16 dBm [31]. Using this power constraint, SAR is decreased to 0.00123 W/kg, which is well below the acceptable safety level. Thus, it can be concluded that this implantable radiator is safe regarding electromagnetic exposure.

**Figure 6:**
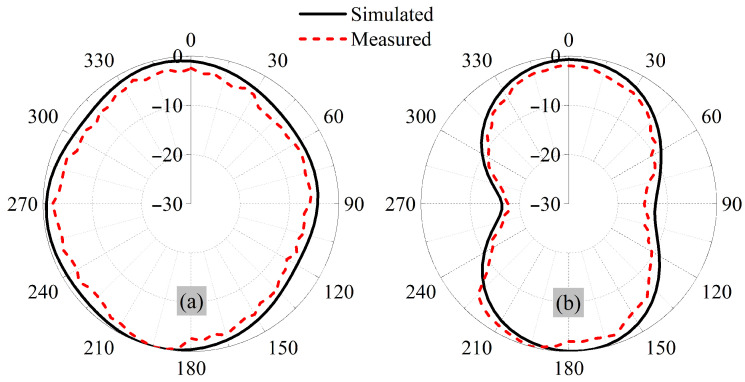
Simulated and measured radiation pattern at 915 MHz (**a**) Phi = 0^∘^ and (**b**) Phi = 90^∘^.

The far-patterns of the antenna are evaluated, where the proposed sensor is placed inside the chamber. The pattern of one element is measured by terminating the other uni and radiation pattern, which is shown in Figure 6. The antenna exhibits near-omnidirectional radiation in both principal planes, which makes it well suited for wireless capsule implant applications. Its orientation changes unpredictably when it moves. With this type of radiation behavior, reliable communication can be maintained regardless of capsule rotation, enabling consistent data transmission in all directions.

At 915 MHz, the simulated value for the peak gain is −25.1 dBi. The gain value from experiments conducted at the same frequency is −26.89 dBi, close to the simulated value.

### 3.1. Link Margin Analysis

A wireless capsule implant moves along the gastrointestinal tract while acquiring physiological data using embedded sensors and imaging modules. The collected information must be reliably transmitted to an external receiver through the integrated implantable antennas. As such, the assessment of the communication ability of the capsule becomes crucial, especially with regard to the support of data communication over a distance.

The range of the transmission can be estimated using the link margin approach. This approach gives an assessment of the reliability of the wireless transmission. In the proposed design, the same antenna is used both as a sensor as well as the radiating antenna. This brings forth the need for conducting the link margin study on the proposed implantable MIMO antenna-sensing system in order to determine the range.

For analysis, it is assumed that the implantable antenna acts as the transmitter as it is used inside the human body. The external dipole antenna, which has a gain value of 2.1 dBi, acts as the receiver antenna. The realized gain of the antenna is −25.1 dBi. Finally, the link margin value is calculated by employing the standard formula for link budget, which includes different losses as well as gains for accurate determination of the transmission range [31].

(3) $$LM=P_{av}-P_{rc}$$

where

(4) $$P_{av}=P_{t}+G_{t}+G_{r}-10log_{10}{\left(\frac{4\times \pi d}{\lambda }\right)}^{2}-N_{\circ }$$

and

(5) $$P_{rc}=E_{b}/N_{\circ }+10log_{10}\left(B_{r}\right)+G_{d}$$

In link budget formulation, the link margin (LM) represents the excess power available at the receiver beyond the minimum level required for reliable data recovery. The parameter $P_{av}$ denotes the available power for data extraction, while $P_{rc}$ refers to the power actually received by the external dipole antenna. The transmitted power from the capsule implant is represented by $P_{t}$ and is selected in accordance with IEEE regulatory limits. The term $G_{t}$ corresponds to the gain of the transmitting antenna, which in this case is the proposed implantable MIMO antenna-sensor.

The required signal quality is defined by the energy-per-bit to noise-density ratio, $E_{b}/N_{0}$, which is set to 9.6 dB for phase-shift keying modulation. Additional system degradation, including implementation and mismatch losses, is modeled using a deterioration factor $G_{d}$ with a value of 2.5 dB. The bit rate, denoted by $B_{r}$, is fixed at two representative values: 78 Mbps and 120 Mbps. These data rates are chosen to meet the requirements of next-generation capsule endoscopy systems, where high-resolution image and video transmission is needed [32].

**Figure 7:**
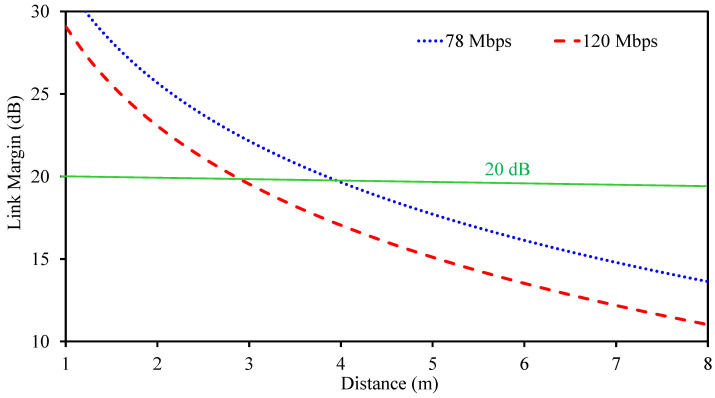
Calculated wireless link margin for the implantable communication link operating at 915 MHz.

To evaluate the communication capability, the separation between the implantable antenna-sensor and the external receiving dipole antenna is gradually increased, and the corresponding link margin is computed. The variation in link margin with distance is shown in Figure 7. Although a link margin of 0 dB is theoretically sufficient to sustain wireless communication, an additional margin is required in practice to account for body losses, antenna misalignment, and environmental uncertainties. Therefore, a conservative link margin threshold of 20 dB is adopted. Under this condition, the proposed MIMO implantable antenna-sensor supports reliable communication over distances of up to 4.1 m at 78 Mbps and 2.95 m at 120 Mbps, demonstrating its suitability for high-data-rate capsule implant applications.

### 3.2. MIMO Channel Parameters

Single-antenna systems are fundamentally constrained in terms of data throughput, as they rely on a single signal path and make minimal use of multipath effects. In contrast, multi-port MIMO antennas can leverage multiple independent signal paths, enabling a substantial increase in channel capacity. Their performance degrades when signals arrive through multiple paths, which reduces data throughput.

In contrast, MIMO antennas are specifically designed to take advantage of multipath environments. By using multiple antenna elements, a MIMO system can support parallel data streams, which significantly improves spectral efficiency. For this reason, evaluating antenna performance in terms of channel capacity is essential.

In theory, maximum channel capacity is achieved when all antenna elements are completely uncorrelated. However, this condition is rarely met in practice due to mutual coupling and limited physical spacing between elements. As a result, some level of correlation is unavoidable. While increasing the number of antenna elements generally enhances channel capacity, this improvement assumes low inter-element correlation, which is difficult to realize in real systems.

Therefore, in practical scenarios, channel capacity depends not only on the number of antenna elements but also on the degree of correlation between them. A system with multiple elements and low mutual correlation yields higher channel capacity than one with many strongly correlated elements. Based on these considerations, the channel capacity of the proposed MIMO antenna system is evaluated using the following formulation.

(6) $$C=\log_{2}\left(\det\left[I+\left(\frac{SNR}{N}\right)HH^{*}\right]\right)$$

In this formulation, *C* denotes the channel capacity and *N* represents the total number of antenna elements in the system. The term *I* is the identity matrix, *H* is the channel matrix, and $H^{*}$ denotes the conjugate transpose of the channel matrix. The channel matrix contains the essential propagation information of the antenna system, including the interaction between its elements. The entries of *H* are typically obtained from the individual radiation characteristics of each antenna element, as described below.

(7) $$H=\left[\begin{array}{cc} h_{11} & h_{12}\\ h_{21} & h_{22} \end{array}\right]=\left[\begin{array}{c} h_{ij} \end{array}\right]\;\mathrm{where}\;i,j\in \left\{1,2\right\}$$

**Figure 8:**
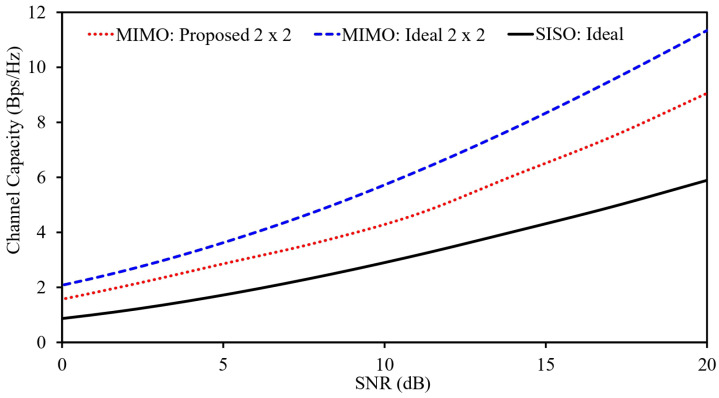
Estimated channel capacity of the wireless link operating at 915 MHz.

$h_{ij}$ includes both amplitude and phase components of the propagation path. In this study, the channel capacities of three configurations are evaluated using (6). The corresponding results are presented in Figure 8.

For the ideal dual-element MIMO case, the analysis assumes fully uncorrelated antenna elements, which represents an upper performance bound. In contrast, the proposed system accounts for the practical correlation between antenna elements. The results show that the ideal dual-element MIMO configuration achieves the highest channel capacity, followed by the proposed MIMO system, while the single-port antenna exhibits the lowest capacity. The reduced performance of the practical system compared to the ideal case is mainly attributed to the nonzero correlation between its elements, which is unavoidable in realistic designs.

A key metric for evaluating MIMO performance is the envelope correlation coefficient (ECC). This parameter quantifies level of correlation between antenna elements and indicates how independently they operate within the system. ECC can be obtained using two main approaches: one based on S-parameters and the other derived from far-field radiation patterns.

In this work, the ECC is calculated using the far-field method, as it provides a more accurate representation of practical radiation behavior. The S-parameter-based approach is generally valid only under ideal assumptions, such as isotropic radiation, which are rarely satisfied in real antenna designs. Therefore, the far-field-based formulation is adopted for this antenna configuration and is expressed by the following equation.

(8) $$\mathrm{ECC}=\frac{{\left|\int \int_{4\pi }\left(A_{n_{i}}\left(\theta ,\phi \right)\right)\cdot \left(A_{n_{j}}\left(\theta ,\phi \right)\right)d\Omega \right|}^{2}}{\int \int_{4\pi }|A_{n_{i}}{\left(\theta ,\phi \right)|}^{2}d\Omega \cdot \int \int_{4\pi }{\left|A_{n_{j}}\left(\theta ,\phi \right)\right|}^{2}d\Omega }$$

In this expression, $A_{n_{i}}\left(\theta ,\phi \right)$ and $A_{n_{j}}\left(\theta ,\phi \right)$ represent the normalized radiation patterns of antenna elements 1 and 2, respectively, while Ω denotes the solid angle over which the integration is performed. Using this formulation, the calculated ECC between the two elements at 915 MHz is less than 0.14, indicating weak correlation and good diversity performance.

Another important diversity metric is the diversity gain, which is directly related to the ECC. It is evaluated using

$$\mathrm{DG}=10\sqrt{1-{\mathrm{ECC}}^{2}}.$$

For the obtained ECC value, the corresponding diversity gain is greater than 9.99 dB, confirming effective diversity behavior of the proposed MIMO antenna system.

### 3.3. Sensing Mechanism

The proposed antenna-sensor is designed to support simultaneous biological tissue sensing and wireless data transmission. The communication capability of the structure has already been validated in the earlier sections through a detailed analysis of its key antenna characteristics. This section focuses on the sensing functionality of the design. First, the physical mechanism responsible for tissue sensing is explained, followed by simulation results that are used to confirm the developed theoretical model.

**Figure 9:**
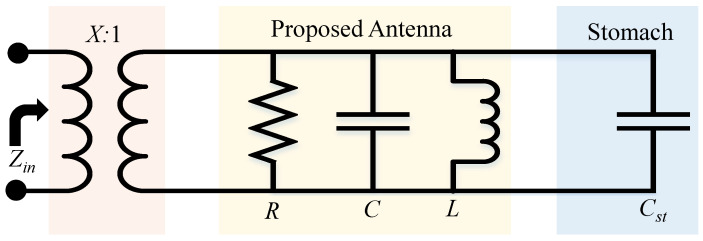
Lumped-element equivalent circuit illustrating the interaction between the implantable antenna and the surrounding human stomach tissue.

When an implantable antenna is positioned close to biological tissue, the surrounding medium effectively acts as a dielectric environment. This interaction introduces an additional capacitive effect. To simplify analysis, the combined antenna–tissue system is represented using an equivalent lumped-element model. The corresponding circuit is illustrated in Figure 9. In this model, the RLC network represents the intrinsic electrical behavior of the antenna, and tissue is modeled as a capacitance, denoted by $C_{st}$.

The term $C_{st}$ denotes the equivalent capacitance arising from electromagnetic coupling with the stomach tissue. This capacitance is determined by the dielectric properties of the tissue together with the permittivity of free space, and can be described by the following expression:

(9) $$C_{st}=\epsilon_{\circ }\epsilon_{st}\frac{K^{\prime }\left(k_{1}\right)}{K(k_{1})}$$

Here, $C_{st}$ represents the additional capacitance introduced by the presence of stomach tissue, $\epsilon_{0}$ is the permittivity, $\epsilon_{st}$ denotes the relative permittivity of the stomach tissue, and *K* refers to the complete elliptic integral. An increase in $\epsilon_{st}$ leads to a corresponding increase in the effective capacitance generated by the tissue loading.

The lumped-element representation of a single antenna-sensor element, including the capacitance associated with the stomach tissue, is illustrated in Figure 9. For clarity, all circuit components are explicitly labeled. Resonant structures operating below their fundamental resonance can be accurately modeled using a purely inductive element, as reported in [33,34,35]. With this approximation, the input impedance of the antenna-sensor system can be expressed as

(10) $$Z=\frac{j\omega L_{equ}}{1-\omega^{2}C_{st}L_{equ}}$$

and

(11) $$\omega =\frac{1}{\sqrt{C_{st}L_{equ}}}$$

The antenna input impedance *Z* is determined by the combined effect of the equivalent inductance $L_{\mathrm{equ}}$, the operating angular frequency ω, and the capacitance $C_{st}$ resulting from the electrical loading of the surrounding stomach tissue. As indicated in (11), the resonance condition of the antenna depends on the combined effect of $L_{\mathrm{equ}}$ and $C_{st}$. Furthermore, (9) shows that the value of $C_{st}$ is directly related to the biological organ’s dielectric constant.

The dielectric properties of stomach tissue, particularly its permittivity, are known to vary with pathological changes such as the formation of gastric lesions and tumor progression [8,9]. As discussed earlier, any change in tissue permittivity modifies the effective capacitance seen by the antenna, leading to a measurable shift in its resonant frequency. This behavior forms the basis of the proposed sensing mechanism, where resonance displacement is used as an indicator of abnormal tissue conditions.

**Figure 10:**
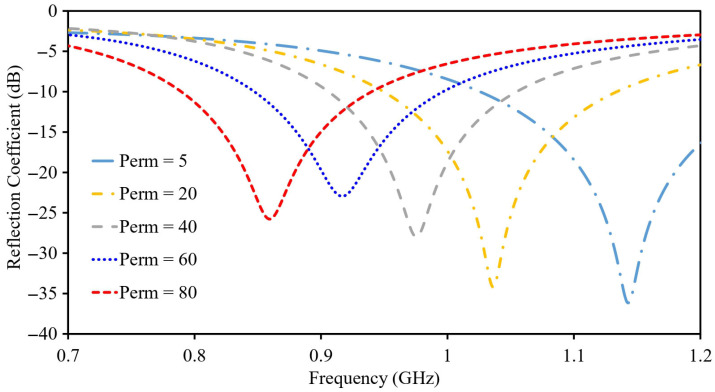
Sensing capability of the antenna-sensor.

Figure 10 presents the simulated $S_{11}$ response of the antenna under different stomach tissue permittivity conditions. A clear upward shift in the operating frequency is observed as the permittivity is reduced. Specifically, the resonance occurs at 1.151 GHz for a permittivity of 5. In contrast, increasing the permittivity to 80 lowers the resonance to 0.855 GHz. This behavior highlights the pronounced dependence of the antenna’s resonant characteristics on the permittivity.

**Figure 11:**
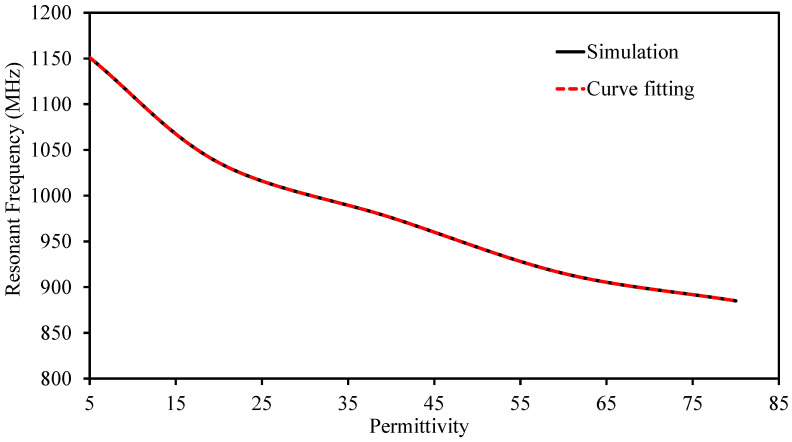
Comparison between simulated data and the corresponding fitted curve.

It maintains good impedance over the entire range of investigated conditions, with the reflection coefficient $S_{11}$ remaining well matched in all cases. Figure 11 shows the simulation results together with the corresponding curve-fitting model. The fitting procedure is performed using the extracted $S_{11}$ data. It should be noted that $S_{11}$ and $S_{22}$ exhibit identical responses.

A polynomial regression model is derived for resonance and dielectric constant, and is given by

$$f=0.0404\;\epsilon_{r}^{2}-6.8225\;\epsilon_{r}+1174.98,$$

where *f* denotes the antenna resonant frequency and $\epsilon_{r}$ is the permittivity of the surrounding tissue. The fitted model shows excellent agreement with the simulation data, yielding a coefficient of determination of $R^{2}=0.99$, which confirms the high sensitivity and repeatability of the proposed sensing mechanism.

Since the primary role of the proposed antenna-sensor is the sending of images from within the body, its key radiation and MIMO performance metrics are evaluated under different sensing conditions. When the surrounding tissue permittivity is set to 5, the antenna exhibits a gain of −21.4 dBi, while an increased permittivity of 80 results in a reduced gain of −28.35 dBi. Despite these variations, strong isolation is preserved across the entire permittivity range, with inter-element isolation remaining better than 27.9 dB in all cases.

The MIMO system is further examined to analyze channel parameters at different sensing states. The envelope correlation coefficient remains below 0.15 for all evaluated permittivity values, indicating low correlation between the antenna elements. In addition, the corresponding diversity gain exceeds 9.9 dB under all conditions. Thus, this system maintains robust MIMO characteristics while performing sensing. It is useful for devices which require concurrent data transmission and tissue monitoring without the need for a separate sensing element.

The proposed implantable MIMO antenna-sensor introduces an integrated solution that combines high data throughput with sensing capability, as summarized in Table 2. In contrast to traditional single-port antenna-based sensors, which suffer from limited communication performance, the use of a MIMO configuration significantly enhances link reliability and data capacity. At the same time, the design overcomes a key limitation of existing implantable MIMO antennas by embedding the sensing function directly into the antenna structure. This unified approach enables efficient wireless communication and also captures physiological changes.

## 4. Conclusions

In this work, a compact implantable MIMO antenna-sensor is introduced for GI applications, serving a dual function of achieving high-speed communication and tumor detection simultaneously. The proposed system comprises two symmetric antenna elements, designed and optimized for 915 MHz, separated by a mere 0.55 mm (edge-to-edge configuration), meeting a compact form factor specification of 11.9 m^3^. Miniaturization is achieved by carefully optimizing the designs using meandered resonators and a high permittivity material for the substrate. Simultaneously, these modifications ensure a reasonable gain of −25.1 dB at 915 MHz, although it is a compact design. A remarkably high isolation level is achieved by appropriately setting the antenna-element distances and optimizing a rectangular-shaped slot on the ground plane, achieving a level above 27.9 dB isolation at both ports. The performance of the designed MIMO system is analyzed through basic channel parameters such as envelope correlation coefficient (ECC), diversity gain (DG), and capacity, at various SNRs. In particular, at an SNR = 20 dB, a channel capacity of 9.05 bps/Hz is attained, proving its capability for high-speed transmission applications. Apart from facilitating fast communication, it serves a further useful purpose as a tissue sensor, detecting resonant frequency variations resulting from changes in tissue permittivities in its vicinity. In particular, it dynamically tracks physiological parameters without using any separate sensing devices, making it a welcome addition during patient monitoring sessions where it retains a high level of isolation, matching, and radiation characteristics consistently throughout tissue-sensing applications too. In these aspects, this proposed compact MIMO antenna-sensor has significant applications in next-generation biomedical devices, making it a prime candidate for GI monitoring applications.

## Figures and Tables

**Table 1:** Frequency-dependent dielectric properties of human stomach tissue, showing variations in relative permittivity and electrical conductivity across the analyzed frequency range.

Frequency (GHz)	0.8	0.85	0.9	0.915	0.95	1
**Conductivity (S/m)**	1.1447	1.1653	1.1867	1.1932	1.2088	1.2316
**Relative Permittivity**	65.362	65.206	65.062	65.02	64.926	64.797
**Loss Tangent**	0.3935	0.37793	0.36429	0.36053	0.35229	0.34167

**Table 2:** Comparison with recently reported implantable antenna designs in the literature.

Ref.	Size (mm^3^)	No. of Elements	Antenna Profile	Freq. (MHz)	Isolation (dB)	Gain (dBi)	MIMO?	Sensing Ability?
[10]	13.2	1	Planar	2450	—	−8.4	No	Yes
[11]	2.97	1	Planar	2450	—	−9.7	No	Yes
[13]	9.01	2	Planar	915	29.7	−24.6	Yes	No
[14]	63.5	2	Planar	2450	22.93	−32.15	Yes	No
[15]	23.6	4	Planar	433	32.6	−28.3	Yes	No
[16]	3375	4	Cubic	2400, 5800	32	−18.5	Yes	No
[17]	307	2	Planar	402	25.6	−26	Yes	No
[18]	434.6	4	Planar	2400	15.9	−15.18	Yes	No
[21]	9	1	Planar	915, 2400	—	−21.8, −19.2	No	No
This work	11.9	2	Planar	915	27.9	−25.1	Yes	Yes

## Data Availability

All the data are available in the study.
